# Assessing the Impact of Thermal Coating Paints on Indoor Temperature and Energy Efficiency in Colombian Caribbean Homes

**DOI:** 10.3390/s25030842

**Published:** 2025-01-30

**Authors:** Frank Florez-Montes, Antonio Martínez-Lengua, Miguel E. Iglesias-Martínez, John Alexander Taborda Giraldo, Eduardo Balvis, Fernanda Peset, Romeo J. Selvas-Aguilar, Juan Carlos Castro-Palacio, Juan A. Monsoriu, Pedro Fernández de Córdoba

**Affiliations:** 1 Faculty of Engineering, Universidad de Manizales, Manizales 170003, Colombia; florez.frank1@gmail.com; 2 School of Exact Sciences and Engineering, Universidad Sergio Arboleda, Santa Marta 470001, Colombia; antonio.martinezl@usa.edu.co; 3 Instituto Universitario de Matemática Pura y Aplicada, Universitat Politècnica de València, Camino de Vera s/n, 46022 Valencia, Spain; miigmar@upv.es (M.E.I.-M.); mpesetm@upvnet.upv.es (F.P.); pfernandez@mat.upv.es (P.F.d.C.); 4 Faculty of Engineering, Universidad del Magdalena, Santa Marta 470003, Colombia; jtaborda@unimagdalena.edu.co; 5 Departamento de Ingeniería de Sistemas y Automática, Escuela Superior de Ingeniería Informática, Universidade de Vigo, Edificio Politécnico s/n, 32004 Ourense, Spain; ebalvis@uvigo.es; 6 Facultad de Ciencias Físico-Matemáticas, Universidad Autónoma de Nuevo León, Av. Universidad s/n, Cd. Universitaria, San Nicolás de los Garza 66455, Mexico; romeo.selvasag@uanl.edu.mx; 7 Centro de Tecnologías Físicas, Universitat Politècnica de València, Camino de Vera s/n, 46022 Valencia, Spain; jmonsori@fis.upv.es

**Keywords:** coating paints, buildings, cool roofs, thermal comfort, sensors, open data, smartphones

## Abstract

Thermal coating paints offer a passive strategy to reduce heat gain in buildings, improve ventilation, and lower energy consumption. This study investigates the effectiveness of these technologies by comparing different housing structures and environmental conditions. Specifically, it examines thermal envelope solutions for cool roofs in homes along the Colombian Caribbean Coast. We quantify the thermal impacts using experimental data collected from 120 houses across eight municipalities in the Magdalena Department, Colombia. The research details the technology and analytical methods employed, focusing on thermal reductions achieved through thermal coatings to potentially reduce energy demand. A comprehensive measurement system, incorporating temperature and humidity sensors, is developed to assess the impact of the coatings. Thermal comfort is evaluated according to the ASHRAE 55 standard, with temperature reductions calculated for each house treated with thermal coatings. A methodology is applied to evaluate the thermal reduction between a house with a coating solution versus a house without it. The results show a temperature reduction on a house-by-house basis, from 1.5% to 16%. On average, the results yield a significant 7% reduction in thermal load. Additionally, a mobile application is developed to disseminate the results of this research, promoting the social appropriation of science among the involved communities.

## 1. Introduction

### 1.1. Generalities

Regional climates have a significant impact on the energy consumption patterns of a population. For example, areas characterized by high temperatures and humidity often experience increased use of ventilation and air conditioning systems, leading to increased energy consumption [1,2]. Although this energy consumption varies with the changing seasons, regions near the equator consistently face high cooling demands [3,4].

Energy consumption in warm regions is intricately linked to comfort requirements within enclosed spaces, such as residential and office buildings. Consequently, achieving thermal comfort becomes a primary goal to achieve energy savings. Thermal comfort is defined as an individual’s perception of temperature and humidity, among other factors. Parameters such as temperature, relative humidity, air velocity, clothing types, and metabolism are recognized factors that affect thermal comfort as described by ISO-7730 and ASHRAE standards [5].

To achieve thermal comfort within enclosed spaces, two main strategies are commonly employed: active and passive [6,7]. Active strategies involve the use of air conditioning systems, which can significantly increase energy consumption. However, through precise control and technological innovations, it is possible to reduce energy consumption. For example, in a study [8], it was reported that by implementing a model predictive control system with adaptive machine learning for the cooling system, significant reductions of 58.5% in cooling energy and 36.7% in the consumption of air conditioning power were achieved compared to the original control methods in the case studies.

In recent research, various innovative approaches have been explored to optimize energy consumption and enhance thermal comfort. For example, the research in reference [9] applied a deep learning algorithm to regulate the energy consumption of fans and air conditioners in a classroom, effectively managing aspects of comfort, including thermal comfort and indoor air quality, for 72 occupants. Remarkably, this optimization resulted in energy savings of approximately 19%, alongside additional health benefits associated with improved ventilation. Similarly, another study [10] utilized predictive control models to dynamically determine temperature set points in spaces with varying occupant distributions. This approach led to substantial energy savings, with a maximum reduction of 21%.

While active strategies like the ones mentioned above are valuable, passive strategies have also gained prominence in the quest to reduce cooling requirements and maintain thermal comfort [11,12,13,14]. One such passive strategy is the implementation of green roofs, photovoltaics, and cool roofs. Green roofs involve the integration of vegetation on exterior walls and rooftops. A simulation conducted in a commercial building in Cyprus, situated in the Eastern Mediterranean region, reported heating energy savings ranging from 6% to 13% [15].

Furthermore, various studies on green roofs have yielded different outcomes depending on geographical location. For instance, research [16] conducted on different green roofs showed variable results based on geographical positions. In warm seasons, cities like Tenerife experienced thermal reductions between 1% and 11%, Seville between 0% and 11%, and Rome between 2% and 8% [16].

Photovoltaic (PV) roofs and thermal coating solutions are becoming increasingly popular as strategies for energy efficiency and comfort enhancement in buildings. Photovoltaic roofs offer numerous advantages, such as local electricity production and shading for various surfaces [17,18,19]. Studies have shown that the success of energy reduction strategies with photovoltaic panels depends on the size of the PV panel system and the availability of roof space. This, in turn, affects thermal reduction, energy production, and carbon emission savings [20].

Photovoltaic systems are instrumental in achieving Zero Energy Building (ZEB) classification, signifying a balance between energy consumption and production [17]. The potential for ZEB classification by utilizing rooftop photovoltaic systems has been explored, with considerations for building shape and height. In residential buildings, the use of solar panels alone has been reported to reduce energy consumption by up to 45% [20]. In warm climates, roofs contribute significantly to the cooling load of buildings, making cool roofs and coating solutions particularly valuable. Research in regions like the Colombian Caribbean, the Mediterranean climate zone of Algeria, and other locations has demonstrated the potential of coating solutions in reducing cooling requirements [17,18,19].

However, the effect of cool roofs can be challenging to model and quantify as exemplified in [14] and [21], where experiments are designed to estimate thermal reductions in enclosed spaces. Finally, it is necessary to explain that the Colombian Caribbean region is a territory characterized by high temperatures throughout the year, which leads to a constant need for energy expenditure in the form of cooling and ventilation systems. The main objective of this project is to improve the thermal comfort conditions of homes in this region, using individually and combined passive zoning strategies and renewable energies.

This article focuses on the use of paint coating solutions for homes in warm-climate regions. The following sections of this article are organized as follows: Section 2 describes the research sites and general information about the project, Section 3 presents the results of the project, Section 4 outlines a discussion on the results, and finally, Section 5 includes the main conclusions drawn from this study.

### 1.2. Project Description

This article presents the analytical and experimental findings from the research project titled “*Investigation of the effects of climate variability and climate change on water resources, biodiversity, and agricultural activities in Magdalena Department*”. This collaborative project involves various public and private organizations from the Colombian Caribbean coast, with a primary focus on assessing the impact of different zoning technologies on the indoor conditions of residential properties within the Magdalena Department. The overarching goal of this research initiative is to provide critical insights and data for informed decision-making processes related to the mitigation of climate change and the adaptation to climate variability in the region. The project was thoughtfully organized and executed with the following key objectives in mind:

- Contribute to the conservation and recovery of ecosystems with a high potential for the adaptation and mitigation of climate change.
- Develop technical conditions for sustainable agricultural and livestock production through profitable adaptation and mitigation actions.
- Promote the conservation and recovery of ecosystems with a high potential for climate change adaptation and mitigation.
- Enhance community resilience by improving living conditions and public health.

The environmental conditions in the Magdalena Department are characterized by high temperatures and relative humidity, which require the use of ventilation and air conditioning (HVAC) systems. However, the excessive reliance on these technologies to improve the thermal conditions of the housingthe housing results in significant energy consumption. The region faces challenges due to a growing population and increased energy consumption, particularly in the context of climate change. This increasing demand for energy resources not only strains natural resources, but also imposes continuous economic costs. Although there is a growing awareness of the urgent need to address the impact of high energy costs in buildings, there is a notable lack of concerted efforts to mitigate these challenges. To address this issue, our research explores two innovative technologies aimed at improving thermal conditions in various communities within the Magdalena Department, while minimizing energy consumption: solar roofs and cool roofs. These technologies involve the installation of photovoltaic systems on rooftops and the application of a specialized coating solution to the exterior surfaces of residential buildings. Although some properties benefit from both technologies, this article focuses mainly on houses equipped with the coating solution.

The project involved 120 households, with an equal number distributed in eight participating municipalities. These households were selected from a low-income housing program initiated by the national government, ensuring that all homes adhered to a standardized design and utilized identical construction materials. Within each municipality, the sample included five houses treated with thermo-insulating paint, five houses equipped with both thermo-insulating paint and solar panels, and five control houses that remained unmodified for comparative analysis.

### 1.3. Description of the Municipalities and Populations

**Figure 1:**
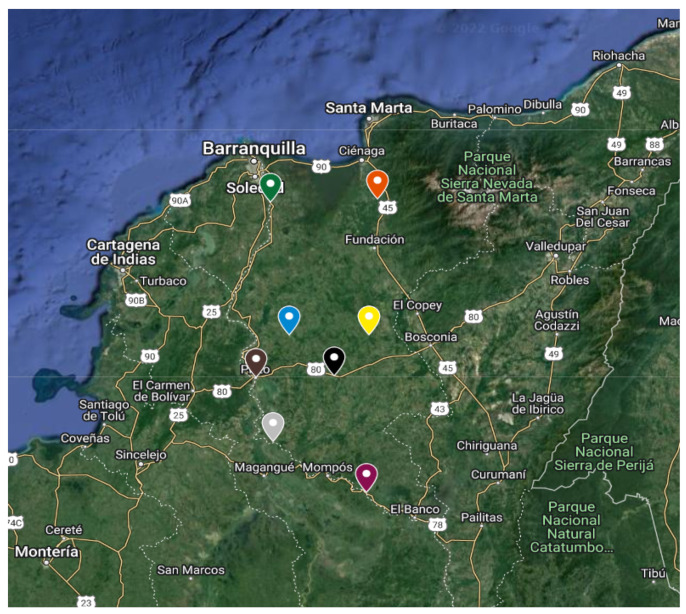
Location of the municipalities participating in the project.

Figure 1 provides a geographical overview of the towns participating in our project, each designated by a unique flag. Sitio Nuevo is marked with a green flag, Zona Bananera with orange, Chibolo with blue, San Ángel with yellow, Platón with brown, Santa Bárbara de Pinto with gray, and Nueva Granada and Guamal with black and purple flags, respectively.

Table 1 presents more detailed information about the participating communities. While these communities share geographical and cultural similarities, their population density provides valuable insights into the characteristics of the municipalities included in the project.

The selected houses were situated within socioeconomic strata 1 and 2. Researchers meticulously selected these properties to minimize the impact of external environmental factors, such as shade cast by trees or the presence of nearby non-residential structures. Each house, regardless of whether it received any zoning technologies, was outfitted with a comprehensive system for monitoring environmental conditions.

The installation of these zoning and metering technologies began in December 2019, and culminated in June 2021, although data collection continued until October 2021. The coating solution is a commercial product, protected by patent secrets; however, the available physical properties are shown in Table 2 [22].

## 2. Materials and Methods

### 2.1. Technology Implementation

**Figure 2:**
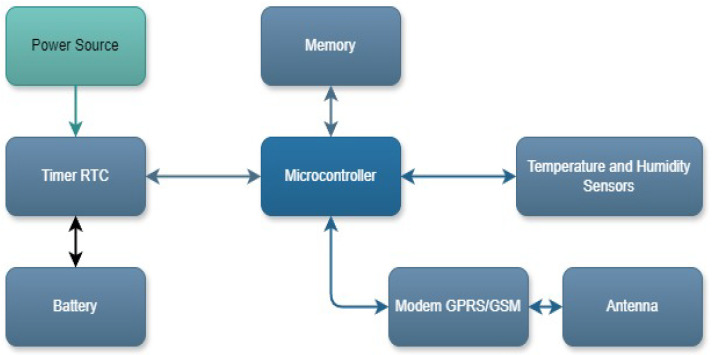
Conceptual design for environmental measurement system.

To assess the impact of zoning technologies, we developed a monitoring system to record indoor temperature and humidity, as well as outdoor ambient temperature for each house. Figure 2 illustrates the conceptual design of this measurement system, which comprises a microcontroller, memory unit, internal battery, geographic positioning modules, environmental sensors, and transmission components. The system is powered by a 12 V adapter and utilizes a quadband modem to establish a GPRS/GSM connection, ensuring compatibility with Colombian mobile operators and supporting the following frequency spectrum: 850/900/1800/1900 MHz. Table 3 includes the key features of the designed datalogger.

**Figure 3:**
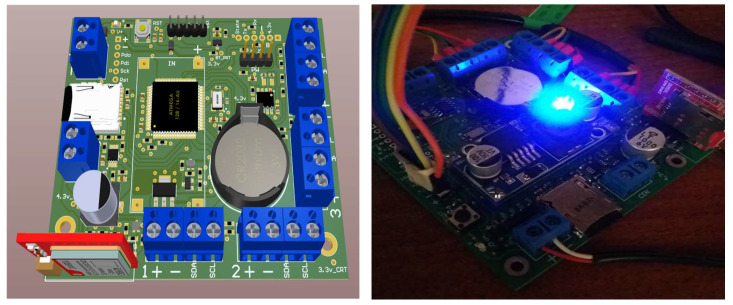
Design and implementation of the recording system. Design in the left image and prototype built in the right image.

The core of the recording system is a Raspberry Pi 3B+ card, which operates continuously whenever power is available. In cases where power is interrupted, the recorded data are temporarily stored in the internal memory and transmitted when power is restored. Figure 3 illustrates the project’s PCB, designed and fabricated. The left panel presents a 3D rendering of the board, while the right panel showcases the assembled board undergoing initial testing. Altium Designer was employed for the PCB design.

**Figure 4:**
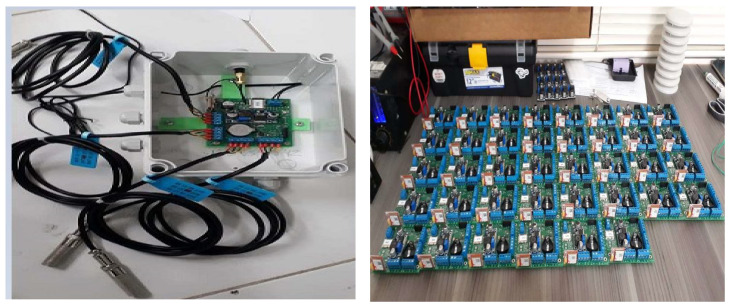
Cards and sensors for installation, image on the left with a fully equipped card ready for installation. The image on the right shows a card bank without sensors.

The system supports multiple temperature and humidity sensors and is enclosed in a protective plastic box, which also houses the power adapter, internal memory, clock, and battery. Four temperature and humidity sensors are connected to the cards, and data are sampled at a frequency of one hour. The collected data are stored in both RAM and external memory, and information is transmitted to the server every two hours. In Figure 4, you can see the recording system along with the sensors and the protective box. A total of 120 logging systems were constructed, with one for each participating house.

**Figure 5:**
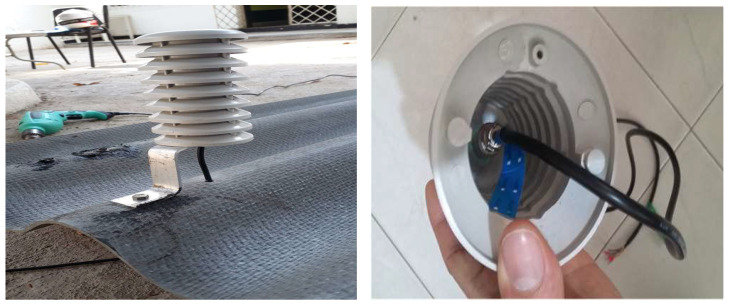
Shield for exterior sensors and their possible position on the roof tile.

The sensors used are SHT20 sensors, which include both a temperature and a humidity sensor. The humidity measurement technology is capacitive, while temperature is measured using bandgap technology. These sensors communicate with the datalogger via the I2C interface, allowing for cable lengths of up to 10 m between the datalogger and the sensors. Inside each house, two SHT20 sensors are installed, while two additional sensors are placed outside and protected by a weather shield, as shown in Figure 5.

### 2.2. Datalogger Operation

The datalogger is designed for low energy consumption and operates in a standby state until it initiates the process of data acquisition, verification, and transmission. The operation of the datalogger device can be summarized in the following stages:

- Acquisition and digitizing: At the specified time, the datalogger sends a command to the humidity and temperature sensors to initiate a new reading.
- Reading: The datalogger communicates with the sensors to collect the measurements.
- Verification: The datalogger validates the obtained measurements, discarding erroneous data, and repeating the reading process if necessary.
- Storage: The data are stored in both external memory and integrated RAM. Following storage, the datalogger returns to the standby state.
- Sending: At the designated time, the datalogger activates the GPRS/GSM modem, prepares the measurements, calculates the CRC, and transmits the data to a server. It then returns to the standby state. If data transmission fails, the data remain stored until a new attempt is made.

**Figure 6:**
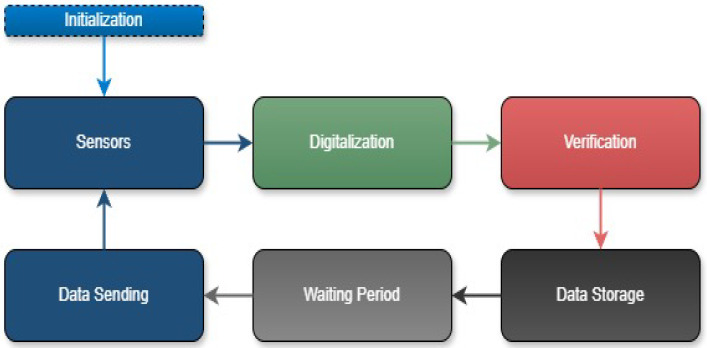
Block diagram of datalogger operation.

The steps described above are summarized in Figure 6.

### 2.3. Research Data Management

The data generated during the research are of two types: (i) data verified with a datalogger, which cleans the raw data coming directly from the sensors installed in the houses, both with and without coating, and the (ii) calculated data. The raw data are not stored or made available, as they contain errors that could bias subsequent research. Due to their ‘dirty’ nature, they are discarded to prevent the experiment from being reproduced with errors.

Both types of data, (i) and (ii), are protected under intellectual property laws. Specifically, the ‘sui generis’ right applicable to databases with manipulated data grants the rights holder the ability to release them or not. Since these are research data, the principle of the European Union, “as open as possible, as closed as necessary”, is followed [23].

Given this research commitment to scientific advancement, some data will be made available in the future once their full potential has been exploited, in order to comply with the FAIR principles (findable, accessible, interoperable, and reusable), as well as Spanish and Colombian regulations. To achieve this, the data will be deposited in a repository that accepts such data, establishing reciprocal links from the articles to the databases. The institutional repository of Universitat Politècnica de València, RiuNet http://riunet.upv.es/, and a general repository, ZENODO http://zenodo.org/, are planned to be used. In addition, a mobile application has been developed to disseminate the project’s results, fostering the social appropriation of science within the involved communities.

### 2.4. Methodology

The effectiveness of the coating solution is evaluated by analyzing temperature data. A single house within a municipality, featuring the cladding solution applied to its exterior surfaces, is compared to all other houses in that municipality that do not utilize zoning technology. Temperature comparisons are performed individually between the house with the coating and each of the houses without cladding. The percentage temperature difference is calculated for each comparison. Subsequently, the average of these individual percentage differences is calculated to quantify the overall thermal reduction achieved by the coating solution compared to the other houses in the municipality. This methodology is adopted to mitigate the influence of individual house-specific factors. For instance, in some locations, natural elements such as trees provide shade, reducing the interior temperature, while in other houses, roofing or wall materials may lead to higher internal temperatures.

**Figure 7:**
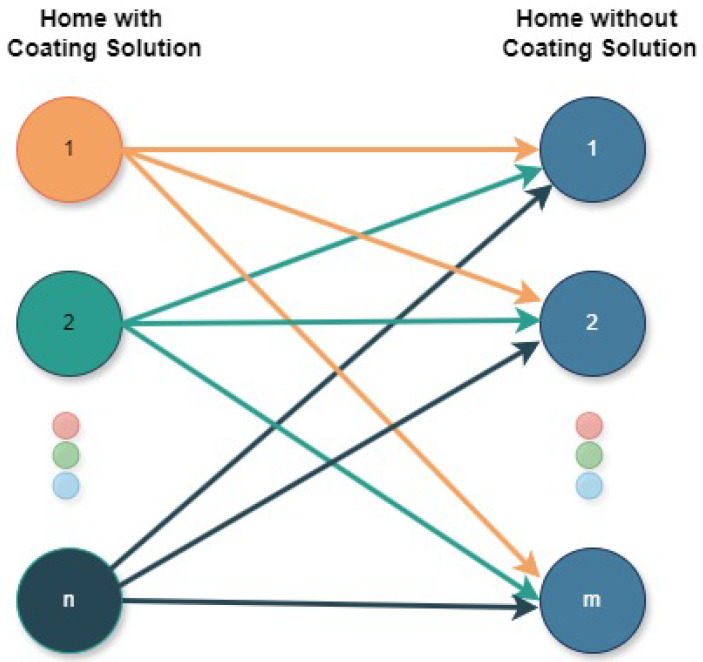
Comparison methodology.

Figure 7 illustrates the correlation between the houses established by the methodology.

To accurately assess the thermal reduction achieved by a coating solution, it is essential to compare the internal temperature of the treated house to a control house without any modifications. Comparing to a house with inherently high internal temperatures, such as a poorly insulated house or one with excessive solar gain, would artificially inflate the perceived thermal reduction provided by the coating. In contrast, comparing to a house with existing shading elements, such as trees or awnings, could mask the true effectiveness of the coating solution, as these elements already contribute to reduced internal temperatures.

The methodology employed ensures a realistic assessment of the impact of the coating solution on a house when compared to all others. To calculate the thermal reduction (TR), we use Equation (1), where Tcs represents the internal temperature of a house with a coating solution, and Ts represents the internal temperature of a house without the solution:

(1) $$TR=\frac{\sqrt{\sum {\left(T_{cs}-T_{s}\right)}^{2}}}{\sqrt{\sum {\left(T_{s}\right)}^{2}}}\times 100\%$$

In addition, we can have as a means of improvement the possible use of artificial intelligence tools and the wavelet transform. These elements could be considered for refinement of the initial data and the use of AI to fill in missing data. This approach will be explored in future research aimed at achieving a more accurate and efficient comparison, ultimately leading to results of higher quality and scientific relevance.

Finally, to guarantee the comfort sensation, the ASHRAE 55 standard comfort psychrometric chart is used.These graphics illustrate the concentration and variation in temperature measurements [5,24].

**Figure 8:**
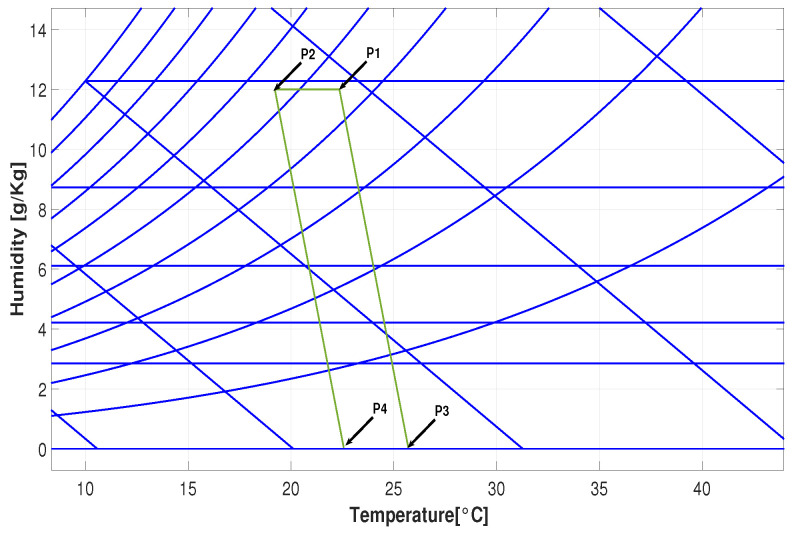
Comfort zone defined for this region.

The ASHRAE 55 standard provides a framework for determining the satisfactory relationship between humidity and temperature [5,25]. The comfort zone shape is a parallelogram, defined by calculating the coordinates specified in Figure 8.

The points $P_{1}$ and $P_{2}$ are determined using Equations (2) and (3), respectively. The thermal insulation provided by the clothing (clo) is set at 0.57, representing typical clothing for the region, specifically, pants and a short-sleeved shirt. The representative temperatures for the region are selected as follows: $T_{P_{1}-sup}=$ 26 °C, $T_{P_{1}-inf}=$ 24 °C, $T_{P_{2}-sup}=$ 23 °C and $T_{P_{2}-inf}=$ 20 °C. We have the following:

(2) $$P_{1}=\frac{\left(clo-0.5\right)*T_{P_{1}-inf}+\left(1-clo\right)T_{P_{1}-sup}}{0.5}$$

(3) $$P_{2}=\frac{\left(clo-0.5\right)*T_{P_{2}-inf}+\left(1-clo\right)T_{P_{2}-sup}}{0.5}$$

Points $P_{3}$ and $P_{4}$ are determined using Equations (4) and (5). The constant Im, as defined by the standard, is set to 0.43. The Lewis Ratio constant (LR) is LR=21.7054 °C/hPa, and the skin moisture (*w*) is approximated as w=0.03:

(4) $$P_{3}=P_{1}-12*w*Im*LR$$

(5) $$P_{4}=P_{2}-12*w*Im*LR$$

## 3. Results

The first result obtained in the project is the construction of a database with environmental and internal data of the houses. The environmental data are studied below.

The geographical proximity of Colombia to the Equator results in challenges for nationalizing processes across its territory. The Magdalena Department, located along the coast, is susceptible to meteorological phenomena such as typhoons or hurricanes. Typically, daytime temperatures average above 30 °C, with nighttime temperatures approaching 25 °C. However, daily averages can exhibit significant variations. In our research, we observe daily averages ranging from 31.3 °C to 26.7 °C within the same month. These variations can fluctuate by up to 15% in daily means, making it challenging to analyze the same house over time on different days.

**Figure 9:**
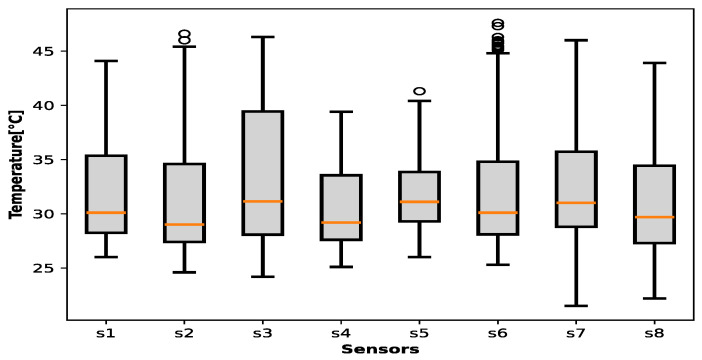
Environmental temperature in all municipalities.

**Figure 10:**
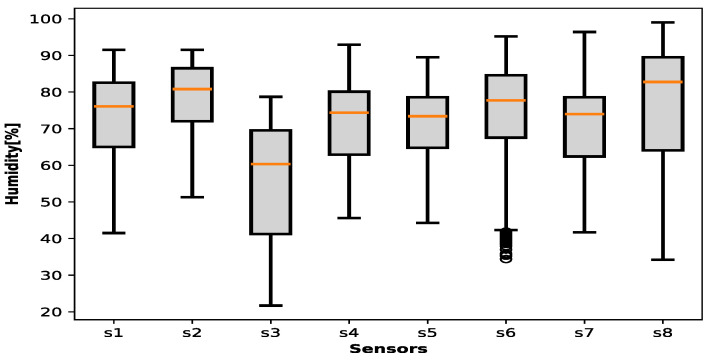
Environmental humidity in all municipalities.

Figure 9 and Figure 10 contain the ambient temperature data collected during the entire project for all houses and municipalities, where the sensors are distributed as follows:

- s1: Sitio Nuevo → 591 registers;
- s2: Zona Bananera → 679 registers;
- s3: Sabanas San Angel → 188 registers;
- s4: Chibolo → 199 registers;
- s5: Santa barbara de pinto → 931 registers;
- s6: Nueva Granada → 1067 registers;
- s7: Guamal → 876 registers;
- s8: Plato → 1128 registers.

Figure 7 demonstrates that temperature across the majority of municipalities exhibits a concentrated distribution between 28 °C and 35 °C, with a median value approximating 30 °C. The dataset exhibits a limited number of observations exceeding 25 °C or falling below 40 °C. In a similar vein, Figure 8 illustrates that humidity levels across the municipalities primarily fall within the 65–85% range, with median values centered around 75%. Nevertheless, instances with humidity exceeding 90% or falling below 50% are observed. These visual representations suggest the presence of potential outliers within the data, necessitating a data preprocessing stage to ensure data quality and accuracy.

Another challenge encountered with the data is the disparity in the number of records collected from each municipality. This is mainly caused by power outages, which are common in some areas and hinder direct comparison. As a result, temperature and humidity were not recorded simultaneously in all homes and municipalities on the same days. To address this issue, a methodology for analyzing the data is proposed. However, the recorded experimental data agree with the theoretical information about the environmental conditions of the region.

To mitigate errors caused by weather variability, estimates and comparisons are conducted on the same day to ensure consistent exposure to environmental conditions for all houses. Before comparing the results for the different houses, it is verified that the recorded environmental conditions are not statistically different. A Student’s *t*-distribution is used for this purpose, generating a *p*-value of 0.03, which leads to the rejection of the hypothesis that the environmental data are different.

**Figure 11:**
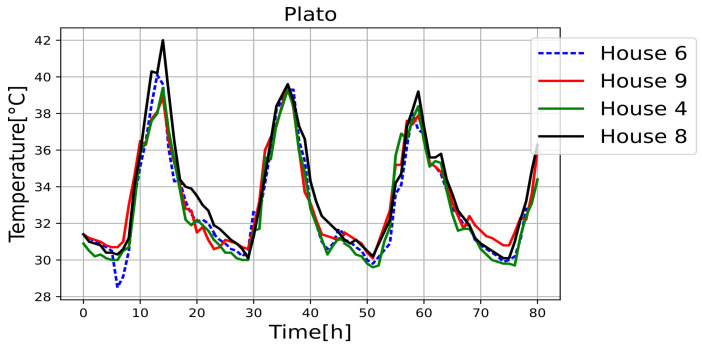
Internal temperature in houses 4, 6 (painted), 9, and 8.

**Figure 12:**
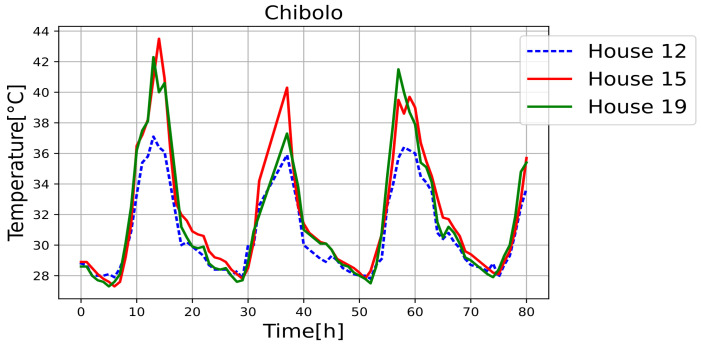
Internal temperature in houses 12 (painted), 15, and 19.

**Figure 13:**
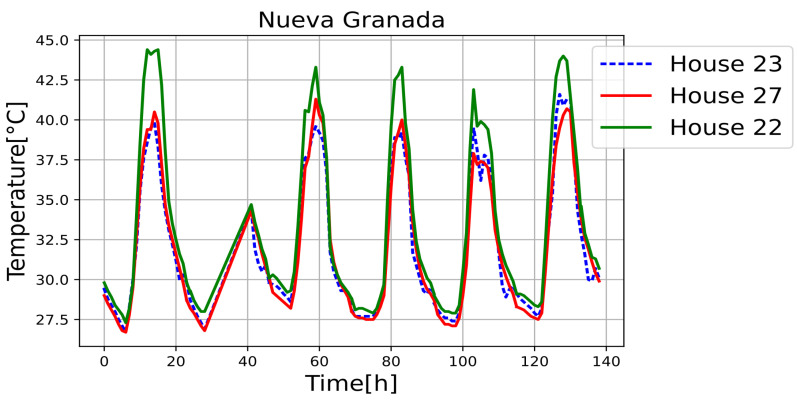
Internal temperature in houses 22, 23 (painted), and 27.

**Figure 14:**
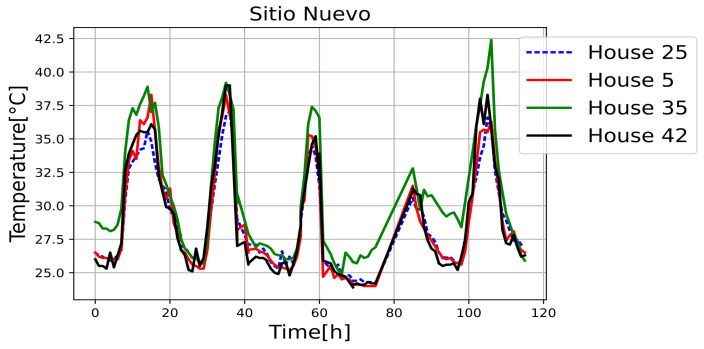
Internal temperature in houses 5, 25 (painted), 35, and 42.

**Figure 15:**
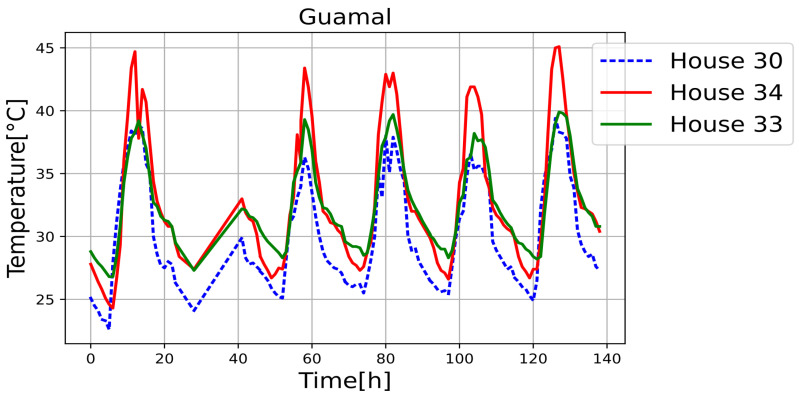
Internal temperature in houses 30 (painted), 33, and 34.

**Figure 16:**
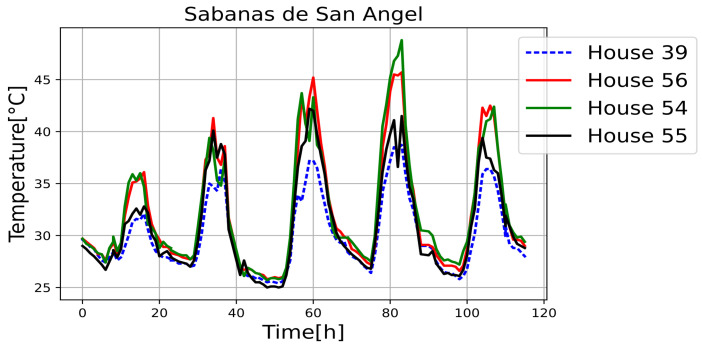
Internal temperature in houses 39 (painted), 54, 55, and 56.

**Figure 17:**
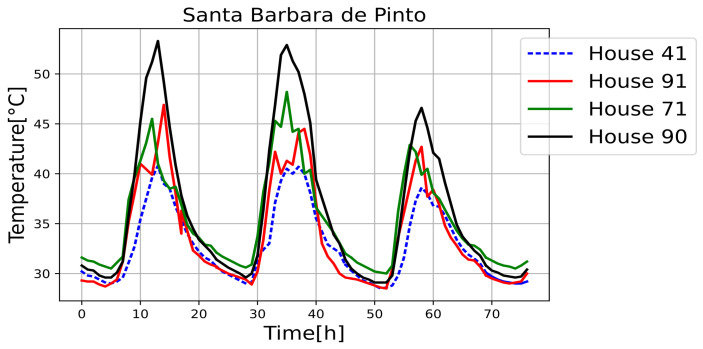
Internal temperature in houses 41 (painted), 71, 90, and 91.

**Figure 18:**
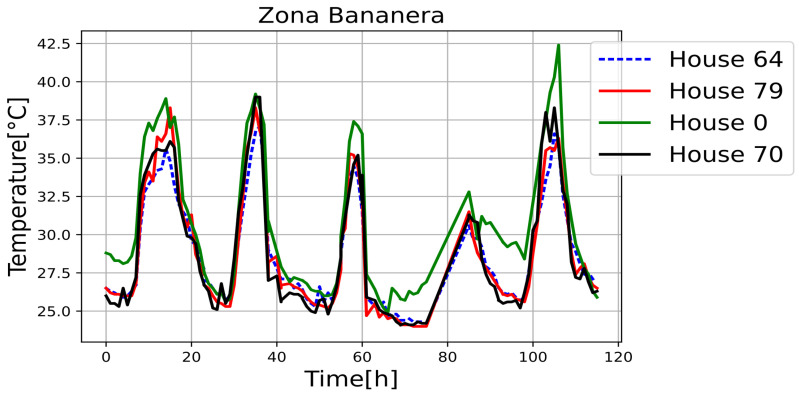
Internal temperature in houses 0, 64 (painted), 79 and 70.

Figure 11, Figure 12, Figure 13, Figure 14, Figure 15, Figure 16, Figure 17 and Figure 18 illustrate the stark differences from one day to the next within the same month. Each figure depicts indoor temperatures in different houses on various days in June 2021, with each figure corresponding to a specific city. In each case, a house with a thermal coating solution is compared to houses without any zoning technology. The blue line in each figure represents the temperature of a house with the thermal solution. The effects of the thermal coating solution are calculated by comparing painted and unpainted houses, subject to the following conditions:

- Temperature reductions are considered only during the daytime period (from 6:00 to 18:00).
- Comparisons are made if there are temperature records for the same day in both houses.
- There must be more than 20 records in a day to enable a comparison.
- Outliers must be fewer than 3 in a single day for comparison.
- For comparison, two data points must be taken less than one hour apart.

The constraints established for this project are essential for addressing the various challenges encountered during data collection and analysis. The database presents a non-uniform structure, characterized by missing data points and inconsistencies in the time series due to frequent power outages. These power outages, a common occurrence in this geographic region, lead to sudden interruptions in data collection, resulting in erroneous data points. These outliers are identified and subsequently removed during the initial stages of data analysis.

**Figure 19:**
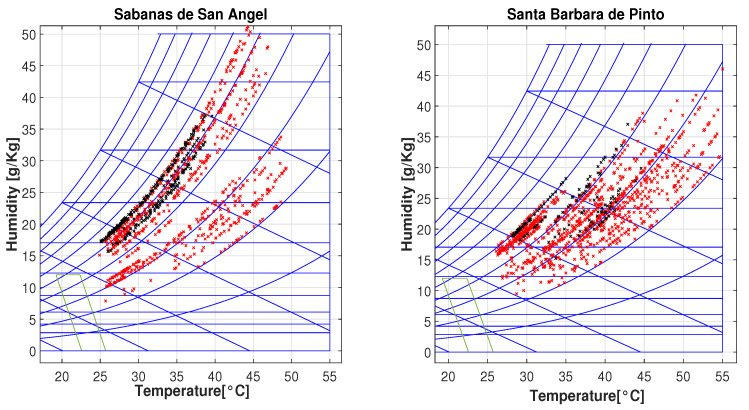
Psychrometric chart for Sabanas de San Ángel and Santa Barbara de Pinto.

The primary objective of the project is to assess the impact of the passive zoning technology on indoor temperatures in residential homes. This article specifically focuses on coating solutions. However, indoor temperature is just one aspect of thermal comfort, which can be evaluated through humidity and temperature measurements. Figure 19 displays a psychrometric chart with a comfort zone for two sample villages, Sabanas de San Ángel and Santa Barbara de Pinto, with black and red dots representing temperature and humidity measurements in houses with and without coating solutions, respectively.

**Figure 20:**
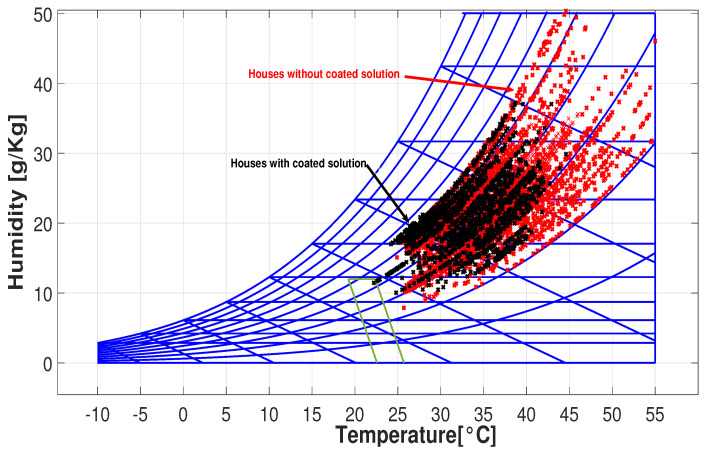
Psychrometric chart for all towns.

Under natural ambient conditions, none of the houses fall within this comfort zone. However, homes with coating solutions are closer to achieving it, indicating that they would require less energy from HVAC systems to reach the comfort zone. Notably, houses with coating solutions (black dots) show lower maximum internal temperatures compared to those without (red dots). This implies reduced cooling requirements for houses equipped with zoning technology. Figure 20 displays temperature and humidity measurements for all houses across different cities. In this figure, you can observe that measurements for houses with coating solutions (black dots) are mostly within the range of 25 to 40 °C), while those without (red dots) exhibit a wider dispersion, sometimes exceeding 50 °C).

## 4. Discussion

Temperature reductions are considered indicative of cooling energy savings, as they lead to the reduced usage of ventilation and cooling systems. The savings for each house are calculated by averaging the temperature reductions across unpainted houses.

**Figure 21:**
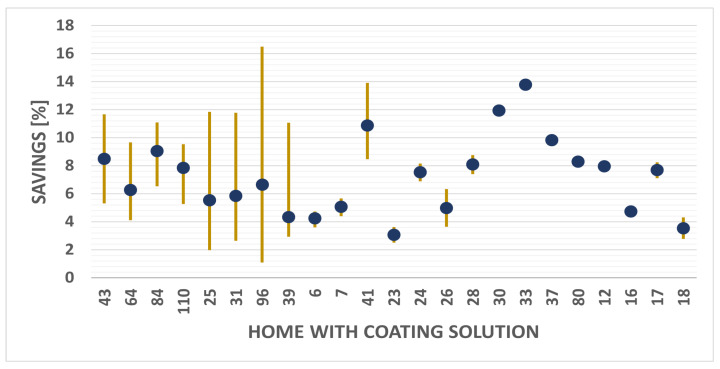
Temperature savings for houses with coating solutions.

Figure 21 illustrates the savings for each house in the study. The blue dots represent the calculated averages, while the yellow line represents the thermal reduction range of the comparison. This methodology prevents the overestimation of results. For example, house number 96 achieved reductions of 16% and 1.5%. Focusing on a single value would not accurately reflect the overall impact of the coating solution. The average energy savings for all the houses in the study amount to 7.19%.

These results are consistent with previous estimates and research. In the [26] research, savings range from 2.1% to 9.13%. In another research on solar roofs [27], the energy savings due to temperature reduction reach 17%. In a research study on cool roofs in Australia, the savings are as high as 8% in residential buildings [28]. All these results are in line with our estimate of 7.19%, and are very close to the calculations developed in our own previous research [29,30,31]. It is important to note that while coating solutions offer benefits, they may not entirely replace the need for ventilation and air conditioning systems but can effectively reduce their usage. Furthermore, the thermal reductions achieved can be enhanced when combined with other zoning technologies such as green roofs and photovoltaic systems [32,33,34]. In future research, it will be valuable to analyze houses equipped with both coating solutions and photovoltaic panels. This analysis should also consider the energy production from photovoltaic systems and their potential economic advantages for the community.

## 5. Conclusions

Addressing the challenges of climate change is a formidable undertaking. To tackle this issue, a pilot project was initiated in Magdalena Department, employing coating solution paints and photovoltaic systems to reduce cooling requirements in diverse communities across the region. This pilot initiative, titled ’Investigation of the effects of climate variability and climate change on water resources, biodiversity, and agricultural activities in Magdalena Department’, encompasses a range of communities, including Sitio Nuevo, Zona Bananera, Chibolo, San Angel, Plato, Santa Barbara de Pinto, Nueva Granada, and Guamal.

Throughout the project’s implementation, a total of 120 measurement cards were manufactured and installed in various households within the participating communities. These measurement cards were equipped with SHT20 sensors to collect data on temperature and humidity. Some households were equipped with both the coating solution and photovoltaic systems, while others received only one of these technologies. This article primarily focuses on assessing the impact of the coating solution.

The importance of passive strategies, such as the use of thermal envelopes, is highlighted as a viable solution to improve thermal comfort and reduce energy consumption in low-income housing. These strategies are especially relevant in contexts where resources for active solutions are limited.

The research demonstrates that zoning technologies have a significant effect on reducing internal temperatures in homes. When comparing houses with and without coating, it was observed that the former were able to maintain lower internal temperatures, which not only improves thermal comfort but also reduces the need for cooling systems, contributing to lower energy consumption.

The collected temperature and humidity data were evaluated using the ASHRAE Standard 55 and presented on a psychrometric chart. The analysis revealed that houses with the coating solution consistently maintained maximum temperatures within the range of 40 °C to 45 °C. In contrast, houses without the coating solution experienced maximum temperatures ranging from 50 °C to 55 °C.

To quantify the energy savings related to cooling, the Euclidean norm was employed for each house. The results showed that, on average, houses with the coating solution achieved energy savings ranging from 2.5% to 14%. When considering all houses, the average energy savings amounted to 7.19%.

Despite the difficulties encountered, such as power outages and data irregularities, the methodology employed for data analysis was effective. This highlights the need for robust and adaptable monitoring systems in studies of this type, which can serve as a model for future research in other regions.

Additionally, it is worth noting that combining coating solutions with photovoltaic systems has the potential to further enhance energy savings. Given the region’s abundant solar radiation, which intensifies cooling related energy consumption, future research endeavors should delve into estimating energy savings from individual photovoltaic panels and combined passive zoning strategies.

## Figures and Tables

**Table 1:** Information about the municipalities included in this research.

Municipality	Surface (km^2^)	Population	Geographical Coordinates
Guamal	554	25,058	$9^{\circ }08^{\prime }39^{\prime \prime }\;N\;74^{\circ }13^{\prime }25^{\prime \prime }\;O$
Plato	1500	48,898	$9^{\circ }47^{\prime }33^{\prime \prime }\;N\;74^{\circ }46^{\prime }57^{\prime \prime }\;O$
Nueva granada	843	16,088	$9^{\circ }48^{\prime }04^{\prime \prime }\;N\;74^{\circ }23^{\prime }30^{\prime \prime }\;O$
Santa Barbara de Pinto	497	12,610	$9^{\circ }26^{\prime }07^{\prime \prime }\;N\;74^{\circ }42^{\prime }06^{\prime \prime }\;O$
Nuevo	967	26,777	$10^{\circ }46^{\prime }33^{\prime \prime }\;N\;74^{\circ }43^{\prime }13^{\prime \prime }\;O$
Zona Bananera	443	4944	$10^{\circ }45^{\prime }51^{\prime \prime }\;N\;74^{\circ }09^{\prime }26^{\prime \prime }\;O$
Chibolo	527	16,018	$10^{\circ }01^{\prime }35^{\prime \prime }\;N\;74^{\circ }37^{\prime }17^{\prime \prime }\;O$
Sabanas de San Ángel	1196	11,425	$10^{\circ }13^{\prime }25^{\prime \prime }\;N\;74^{\circ }12^{\prime }56^{\prime \prime }\;O$

**Table 2:** Physical properties of the coating solution.

Physical Property	Value	Unit
Thermal conductivity	0.0556	W/(m·K)
Density	485.4	kg/m^3^
Thickness (recommended)	0.5	mm
UV refractive percentage	82	%
Brookfield Viscosity	38,200	Cp

**Table 3:** Key features of the designed datalogger.

Parameter	Description
Operation voltage	12 VDC
Energy consumption	0.5 W
Sensors	Up to 4 humidity and temperature sensors
Sampling rate	Every 1 h
Shipping frequency	Every 2 h
External memory	256 MB (1 million 200 thousand measurements approx.)
Communication	GPRS/GSM
Sensibility	−106 dBm
Bands	850/900/1800/1900 MHZ

## Data Availability

The data that support the findings will be available in RiuNet or Zenodo at http://riunet.upv.es/ or http://zenodo.org/ following an embargo from the date of publication to allow for commercialization of research findings.
